# Optimising β-Ti21S Alloy Lattice Structures for Enhanced Femoral Implants: A Study on Mechanical and Biological Performance

**DOI:** 10.3390/ma18010170

**Published:** 2025-01-03

**Authors:** Lorena Emanuelli, Melika Babaei, Raffaele De Biasi, Anton du Plessis, Andrea Trivisonno, Francesca Agostinacchio, Antonella Motta, Matteo Benedetti, Massimo Pellizzari

**Affiliations:** 1 INSTM Operative Center, University of Trento, 38122 Trento, Italy; lorena.emanuelli@unitn.it; 2 Department of Industrial Engineering, University of Trento, 38123 Trento, Italy; melika.babaei@unitn.it (M.B.); raffaele.debiasi@unitn.it (R.D.B.); f.agostinacchio@unitn.it (F.A.); antonella.motta@unitn.it (A.M.); massimo.pellizzari@unitn.it (M.P.); 3 BIOTech Research Center, University of Trento, 38123 Trento, Italy; 4 Research Group 3D Innovation, Stellenbosch University, Stellenbosch 7602, South Africa; anton.duplessis@comet.tech; 5 Object Research Systems, Montreal, QC H3C 1M4, Canada; 6 Trumpf Additive Manufacturing Italia s.r.l, 36036 Schio, Italy

**Keywords:** Ti21S, TPMS-gyroid, auxetic re-entrant bow-tie, functionally graded porous structures, quasi-static and fatigue mechanical properties, osseointegration

## Abstract

The metastable β-Ti21S alloy exhibits a lower elastic modulus than Ti-6Al-4V ELI while maintaining high mechanical strength and ductility. To address stress shielding, this study explores the integration of lattice structures within prosthetics, which is made possible through additive manufacturing. Continuous adhesion between the implant and bone is essential; therefore, auxetic bow-tie structures with a negative Poisson’s ratio are proposed for regions under tensile stress, while Triply Periodic Minimal Surface (TPMS) structures with a positive Poisson’s ratio are recommended for areas under compressive stress. This research examines the manufacturability and quasi-static mechanical behaviour of two auxetic bow-tie (AUX 2.5 and AUX 3.5) and two TPMS structures (TPMS 2.5 and TPMS 1.5) in β-Ti21S alloy produced via laser powder bed fusion. Micro-CT reveals printability issues in TPMS 1.5, affecting pore size and reducing fatigue resistance compared to TPMS 2.5. AUX 3.5’s low stiffness matches cancellous bone but shows insufficient yield strength and fatigue resistance for femoral implants. Biological tests confirm non-toxicity and enhanced cell activity in β-Ti21S structures. The study concludes that the β-Ti21S alloy, especially with TPMS 2.5 structures, demonstrates promising mechanical and biological properties for femoral implants. However, challenges like poor printability in TPMS 1.5 are acknowledged and should be addressed in future research.

## 1. Introduction

The use of metallic materials in biomedical applications is driven by their favourable mechanical properties, including strength, corrosion and wear resistance, high fatigue resistance, and thermal and electrical conductivity. Over 70% of implants used worldwide are comprised of metallic materials [1]. For metal implants, especially those employed in load-bearing applications, the primary essential characteristics revolve around enhancing tissue strength without triggering adverse reactions in the host tissue or organ [2]. Decades of medical studies have established titanium’s bioinertness, attributed to its ability to produce an oxide film even in low-oxygen environments [3]. This property helps avoid adverse chemical reactions in body fluid, making titanium and its alloys highly attractive in the production of implants. Titanium’s preference over chromium-cobalt alloys and stainless steel is further reinforced by its mechanical properties, which closely resemble cortical bone, including ductility, corrosion resistance, density, thermal conduction, and an elastic modulus. Moreover, titanium exhibits excellent biocompatibility properties, such as protein adsorption, the formation of calcium phosphate, and osseointegration in live tissue [1,2].

In the orthopaedic field, the metallic biomaterials used for the implant must be biocompatible and characterised by similar mechanical properties of the replaced and/or repaired bone. To avoid the so-called “stress shielding effect” that leads to bone resorption and, consequently, the necessity for further surgical operations, the stiffness of the metallic biomaterial must be close to the one of the human bones (<30 GPa) [4]. Ti-6Al-4V extra-low interstitial (ELI) is a widely used metallic biomaterial thanks to its high strength and corrosion resistance. Nevertheless, due to the long-term harmful effects on patient health of some elements, namely, aluminium (Al) and vanadium (V), in the last years, researchers have been focusing on developing new titanium alloys with a reduction in these harmful elements [5,6,7]. In addition, the stiffness of this alloy is close to 110 GPa, which is still too high compared to that of the bone. Accordingly, actual studies focus their attention on a novel metastable β titanium alloy, namely, Ti21S, which is characterised by Young’s modulus of around 52 GPa with a good strength of around 830 MPa and an extraordinary elongation of 21% [8,9,10].

Human bones exhibit a dual structure, comprising a resilient cortical outer layer and a porous trabecular inner layer. The cortical bone imparts strength, while the trabecular layer facilitates vascularization and nutrient flow essential for bone remodelling. In the context of creating a durable bone implant for prolonged use, two pivotal functions must be fulfilled as a bone replacement. Firstly, the implant must provide robust mechanical support in environments prone to corrosion, ensuring structural integrity throughout its entire duration within the body. This guarantees that the implant remains sturdy and resilient, even in challenging physiological surroundings. Secondly, the implant should prioritise optimal osseointegration to minimise the risks associated with aseptic loosening and reduce the likelihood of revision surgeries. By maximising osseointegration, the implant establishes a strong and enduring connection with the surrounding bone, promoting long-term stability and functionality.

Achieving optimal osseointegration enhances the integration of the implant with the surrounding bone, promoting stability and long-term success in the patient’s skeletal structure [11,12]. The stiffness of bone varies depending on the specific region, with the elastic modulus of cortical bone ranging from 4 to 30 GPa and trabecular ranging from 0.1 to 4 GPa.

The stiffness of the Ti21S alloy remains still too high compared to human bone. To address the stress shielding effect, porous structures can be incorporated into the implant design to reduce its overall stiffness and better match the mechanical properties of nearby bones [13,14]. The additive manufacturing (AM) process, namely, laser powder bed fusion (LPBF), enables the production of complex and intricate lattice structures by selectively melting the powders layer by layer using a laser source [15]. Optimising processing parameters, including power, scan speed, hatch spacing, layer thickness, and chamber environment, is crucial to achieving defect-free components [16]. Without proper optimisation, internal defects such as a lack of fusion, keyhole porosity, and cracks may form. Additionally, manufacturing imperfections resulting from the printing process, such as variations in cross-section, excess material at junctions between struts or ligaments, and strut waviness, can affect the final mechanical response of the lattice structure [10,17,18]. Due to the cyclic nature of the body loads, fatigue is a crucial parameter to be considered in the design of an orthopaedic device. Manufacturing-induced defects are shown to play a major role in the determination of the fatigue life of the lattice component [19]. Among all, partially melted particles, normally found on the AM lattice component surface, are detected as “killer defects” that are able to host crack initiation sites and lead to fatigue failure [20,21]. These porous structures can be classified into two main groups: strut-based and Triply Periodic Minimal Surface (TPMS)-based lattices. Strut-based lattices are composed of a unit cell repeated in space, formed by unidimensional struts converging in nodes according to different topologies [22]. Under external compression loads, lattice structures undergo deformation through stretching, bending, and twisting of struts and ligaments. A formal unit cell categorization in this regard can be provided following Maxwell’s criterion [23]. It is well known in the literature that the mechanical properties of porous metals can be tailored according to the designer’s needs. Actions on the cell topology and the relative density have, for instance, a strong impact on the elastic properties and on fatigue life. Focusing on the latter, strut-based lattices exhibit a fatigue resistance of about 20–30% of the yield strength [24]. Of particular interest are auxetic re-entrant bow-tie structures, which have a negative Poisson’s ratio—interesting when the stress field on the implant promotes its detachment from the bone [18,25,26,27,28]. Furthermore, the auxetic re-entrant bow-tie structure’s pore geometry incorporates acute-angled corners, which, based on the curvature-driven model, serve as the initial point for cell attachment [29,30]. Initiation of cellular growth occurs at the negative-curvature parts of the pores, while cells in the zero-curvature section of the pore await neighbouring points to attain the appropriate curvature. Subsequently, cell expansion proceeds towards the centre of curvature, with the objective of reducing local curvature. Over time, the remaining space gradually fills through tissue formation, advancing toward the centre of the pore. The presence of acute-angle pores serves a dual purpose. Firstly, it aids in cell bridging over the corners by minimising the distance between struts, thereby influencing cell proliferation. Secondly, once the pores transition into a circular shape, the controlled regulation of cell proliferation helps prevent excessive cell occlusion on the scaffold’s surface. This design proved to be advantageous in maintaining the permeability of 3D scaffolds, effectively preventing tissue necrosis within the scaffold’s core [31]. The design parameters of the cell, such as aspect ratio (a/b) and θ angle, influence the Poisson’s ratio and elastic modulus.

Differently, TPMS-based structures, which are defined mathematically, offer several biological advantages including high surface area, minimal surface curvature and good permeability [32,33,34]. The TPMS structure’s large surface area and highly interconnected pore design ensure a large space for cell attachment and migration into the inner parts of the scaffold, facilitating the deposition of the extracellular matrix. The smooth surface of the pores in this design along with the substantial pore size guarantees an efficient permeability rate for oxygen and body fluid, decreases the risk of blockages, extends the time and space for exchanges through the pores, and increases the likelihood of new tissue formation and angiogenesis [35]. Moreover, TPMS-based structures are characterised by a high compression fatigue resistance, found to be 50–60% of the yield stress [36]. The improvement of the fatigue resistance can be attributed to the improved printability and absence of junctions in the TPMS lattice topology [32,37]. TPMS-based lattices can be further divided into skeletal structures and TPMS sheets, with the former exhibiting interconnected porosity, a necessary factor for increasing the permeability of nutrients into the bulk of the implant, and a lower elastic modulus compared to the latter [32,33,34]. The creation of a graded porosity inside the implant, namely, functionally graded porous structures (FGPSs), to mimic the variable porosity inside the trabecular inner part of the bone is of fundamental importance to attain mechanical and biological efficiency in terms of high strength, low stiffness, and improved tissue ingrowth [38,39,40,41,42,43]. The successful formation of a permanent bond between the metal implant and the host tissue relies on effective tissue ingrowth promotion into the implant within a sufficient time frame. Therefore, it is crucial that the pore size is optimised to reduce tissue growth time, facilitate implant fixation, and allow efficient permeability of oxygen, nutrients, and metabolic waste to and from the implant [35]. Scientists have identified an inverse correlation between cell attachment efficiency and implant permeability. As the pore size decreases, permeability diminishes; however, cell attachment increases. Studies have proposed a 300 μm pore diameter as the lower threshold for optimal cell attachment and proliferation in bone substitute applications [31,44,45,46,47,48]. However, the benefits of a larger pore size should not be overlooked, as it increases flow velocity and facilitates cell penetration into the deeper regions of the scaffold. Larger pores provide favourable permeability to the structure, while smaller pores offer a more advantageous curvature that promotes cell proliferation [44]. In various studies involving in vitro or simulated in vivo evaluations of bone cell lines, it has been observed that scaffolds with a pore size of approximately 700 μm demonstrated the highest and most favourable proliferation compared to other pore diameters within the range of 400–1100 μm [44,45,46]. In terms of osteogenic differentiation behaviour, research has shown that in larger pore sizes of approximately 1000 μm, cells are limited to attaching to a single strut, resulting in exposure to stimuli from a very limited surface area. Conversely, in smaller pores, cells have the capability to adhere to multiple struts, leading to a more diverse array of stimuli. However, it is important to note that gene expression on scaffolds with pore sizes smaller than 300 μm is significantly low, sometimes approaching levels observed in nonporous scaffolds [35,44,47,48]. Indeed, a pore size in the range of 300–700 μm is essentially on the side where osseointegration must be guaranteed thanks to the enhancement of cell proliferation and osteogenic differentiation [49,50,51,52].

Considering the diverse biological and mechanical requirements, it is logical to design distinct prosthetic regions dedicated to specific functions. In this endeavour, the versatility of lattice structures proves to be a potent tool, offering design flexibility and the capability to create functionally graded porous structures (FGPSs). However, the production of FGPSs is not without challenges, as the potential discontinuity between layers needs careful consideration, especially with respect to trabecular unit cells. Addressing these issues is crucial for ensuring the seamless integration and effectiveness of such prosthetic designs. Differently, the nature of TPMS structures defined by implicit equations permits to guarantee of continuity inside the gradient [39,53,54,55,56,57]. The effect of the FGPS on the stiffness of the overall structure was studied by S. Ruiz de Galarreta et al. [58]. A mixture rule dependency between the elastic modulus and the density of the different levels is defined with a longitudinally graded porosity as shown in Equation (1).

(1) $$\frac{1}{E}=\sum_{i=1}^{n}k_{i}\frac{1}{E_{i}}$$

Differently, considering longitudinally graded structures, the elastic modulus of the entire structure is dominated by the weakest layer of the FGPSs as shown in Equation (2).

(2) $$E=\sum_{i=1}^{n}k_{i}E_{i}$$

*n* is the total number of layers, *k_i_* refers to the volume fraction of the corresponding layer with respect to the total volume, *E* is the elastic modulus of the FGPSs, and *E_i_* is the elastic modulus of the corresponding layer i.

Metrological and mechanical analyses on Ti21S FGPSs with both auxetic re-entrant bow-tie and TPMS geometries made using laser powder bed fusion were performed by the authors in previous works [18,59]. The printability of the lattice structure, affected by the production process parameters, has an important role in the mechanical response of the structures highlighting the importance of the metrological investigation to accurately predict the mechanical behaviour of the implant [60]. In this study, comparisons between the quasi-static compression mechanical properties, namely, stiffness and yield strength, of a constant single-density structure with the FGPS structure on two different auxetic re-entrant bow-tie re-entrant bow-tie geometries and two TPMS with different unit cell sizes are performed. In addition, considering the aim of the FGPS to merge between an optimal pore size for improved osseointegration and an optimal density to achieve a stiffness close to the one of the human bone, evaluation of the biocompatibility by means of cytotoxicity and metabolic activity tests on the optimal porosity level for biological assessment are conducted. Nevertheless, focusing on the final application of these FGPS lattice structures where the implant undergoes a fatigue load, the fatigue response of the more critical relative density level for each condition is evaluated.

## 2. Materials and Methods

### 2.1. Specimen Design and Preparation

**Figure 1:**
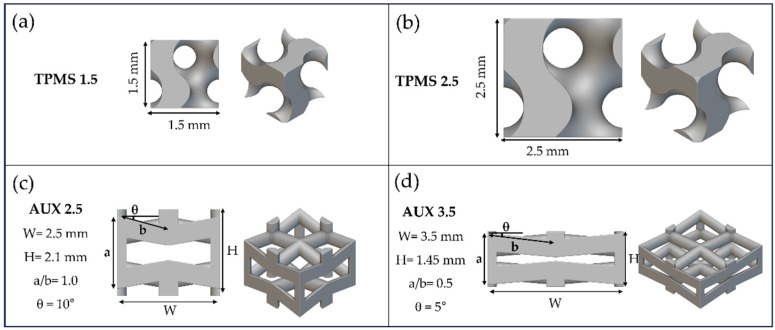
Geometrical details of the (**a**) TPMS 1.5, (**b**) TPMS 2.5, (**c**) AUX 2.5, and (**d**) AUX 3.5.

Two different skeletal-based gyroid TPMSs, namely, TPMS 1.5 (Figure 1a) and TPMS 2.5 (Figure 1b), and two different auxetic re-entrant bow-tie structures, namely, AUX 2.5 (Figure 1c) and AUX 3.5 (Figure 1d), were designed by means of nTop software, V4.2.3 (nTopology Inc., New York, NY, USA).

#### 2.1.1. FGPS Specimens

**Figure 2:**
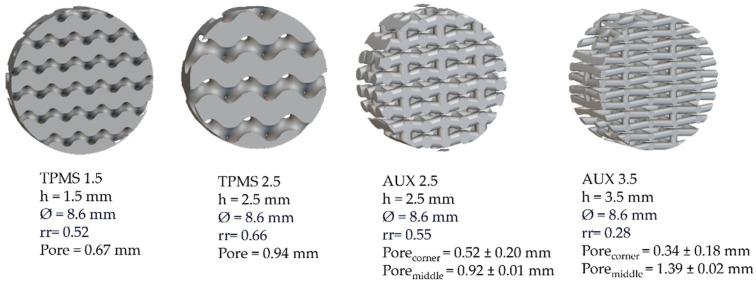
Geometrical details of biological specimens (Bio samples).

Functionally graded porous structures with 3 different relative density levels and single-density specimens with density levels defined for mechanical or biological assessment are designed and printed. Details are summarised in Figure 2.

**Figure 3:**
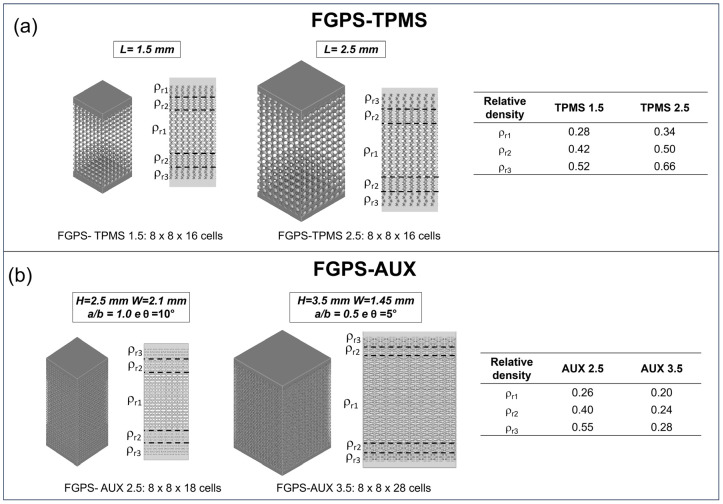
Details of the (**a**) FGPS-TPMS 1.5 and 2.5 and (**b**) FGPS-AUX 2.5 and 3.5.

The FGPSs are designed by fixing the dimension of the lowest relative density level (ρ_r1_ in the middle of the FGPS structure) equal to the dimension of the corresponding single-density specimen designed for mechanical testing. A linear ramp, having a length of 0.2 mm, is generated between the level constants to connect continuously the different porosity levels. Two solid bases with a thickness of 2 mm are added at the bottom and at the top of the structure to evaluate the connection between porous and bulk parts in the implant and to guarantee a flat surface for mechanical testing. The highest (ρ_r3_) and the lowest (ρ_r1_) relative density levels are designed, respectively, to favour osseointegration and to decrease the elastic modulus close to that of the cancellous bone. All CAD parameters are characterised by means of 3D image analysis software ORS-Dragonfly 2022.2 (Comet Technologies Canada Inc., Montreal, QC, Canada). The software measures the different dimensions by means of the wall thickness analysis method, which consists of evaluating the local thickness of the ligament/strut or pore by fitting its volume with the maximum spheres at each location in the 3D structure [18,27,59,61]. Examples of the data obtained using 3D image analysis for TPMS and auxetic re-entrant bow-tie structures are shown in Figure 3.

**Figure 4:**
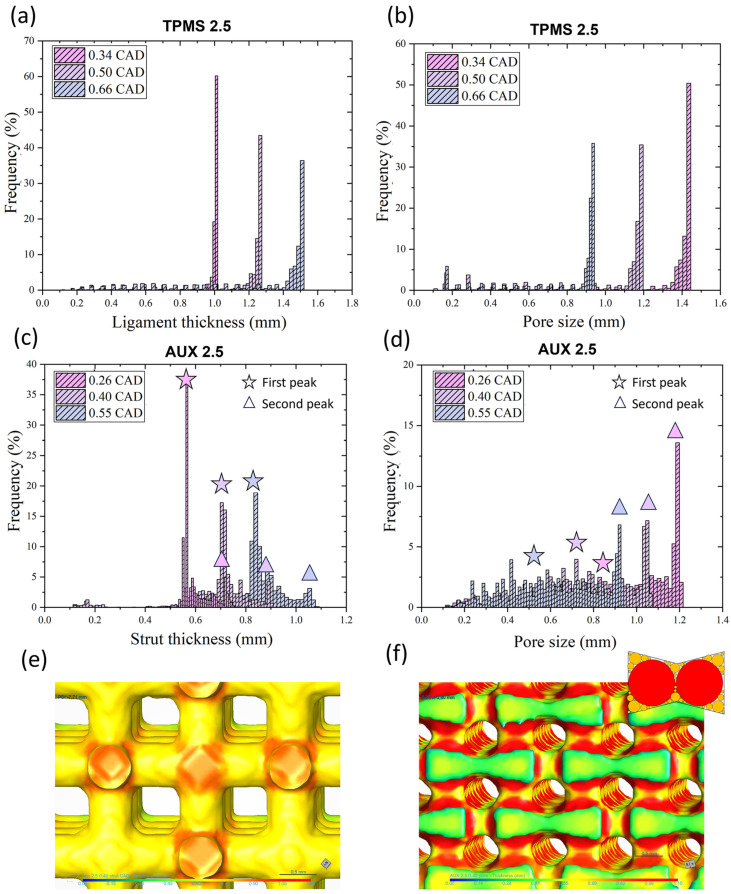
Histograms of the (**a**) ligament thickness and (**b**) the pore size of FGPS-TPMS 2.5 and (**c**) strut thickness and (**d**) pore size of the FGPS-AUX 2.5. (**e**) Mapping of the strut thickness of AUX 2.5 highlighting the struts and nodes. (**f**) Mapping of the pore size to justify the two peaks present in the corresponding histogram.

Considering the designed FGPS-TPMS structures, the data distribution of the ligament (example of TPMS 2.5 in Figure 4a) and of the pore size (example of TPMS 2.5 in Figure 4b) could be well interpolated by means of mode value (the value that occurs most often).

Differently, in the case of the auxetic re-entrant bow-tie structures, two peaks in both strut thickness (example of AUX 2.5 in Figure 4c) and pore size (example of AUX 2.5 in Figure 4d) are detected. The two peaks in strut thickness distribution are ascribed to the strut (first peak) and to the nodes (second peak) as highlighted in Figure 4e. Considering the pore size, the very wide first peak is associated with the small spheres that can fit the corners of the auxetic re-entrant bow-tie structure, and the second peak refers to the biggest pores in the middle of the structure as demonstrated in Figure 4f. All acquired data are summarised in Table 1.

As anticipated previously, the highest relative density (ρ_r3_) in the TPMS structures permits to achieve the pore size in the optimal range for osteointegration, enhancing cell proliferation and osteogenic differentiation [49,50,51,52]. Considering the auxetic re-entrant bow-tie structures, even if the middle pore size is too high even in the (ρ_r3_), the corner pore size results in the optimal range. Indeed, cell attachment and bridging could occur over the corners by minimising the distance between struts, thereby influencing cell proliferation [31,35]. Considering the lowest relative density (ρ_r1_), bigger pores permit to decrease in the stiffness and, therefore, to match it with that of human bone. Nevertheless, a decrease in the stiffness brings about a decrease in the strength and fatigue resistance.

#### 2.1.2. Single Density Specimens

Single-density specimens for quasi-static and fatigue mechanical testing are designed to guarantee an aspect ratio close to 1 as indicated in the Standard ISO 13314 [62]. The geometrical details are summarised in Table 2.

Cylindrical specimens for biocompatibility testing are designed in accordance with the Standard ISO 10993 [63], with their corresponding relative density optimised to enhance osseointegration.

The samples are printed by means of an LPBF machine model TruPrint 2000 (Trumpf Additive Manufacturing Italia s.r.l, Schio, Vicenza, Italy) on a platform measuring 200 mm in an Argon (Ar) atmosphere, with a laser spot size of 80 μm, a layer thickness of 60 μm, a power of 330 W, and a volumetric energy density of 33 J/mm^3^. An optimised scan strategy and the positioning of the samples are adapted depending on the geometries. In detail, auxetic re-entrant bow-tie structures were printed tilted at 90 degrees to permit better manufacturability of the inclined struts. Conversely, the TPMS structures were printed with the porosity gradient parallel to the building direction. A pre-alloyed plasma atomised β-Ti21S powder alloy produced by Pometon (Pometon, Maerne, Vicenza, Italy) was used. It is characterised by a powder size distribution of 20–55 μm, and the chemical composition is summarised in Table 3.

The Mo equivalent (*MoE*) calculated by means of Equation (3) highlights that this alloy is a metastable β Ti alloy since it occurs in the interval between 10 and 30 wt.% [64].

(3) MoE=1.0Mo+0.67V+0.44W+0.28Nb+0.22Ta+2.9Fe+1.6Cr+1.25Ni+1.70Mn+1.70Co−1.0Al (in wt.%)

### 2.2. Metrological Characterization

Three-dimensional (μ-CT scan) metrological characterization is performed using the Nanotom S system (GE Healthcare, Chicago, IL, USA) with an X-ray voltage ranging between 100 and 130 kV, a current of 80–90 μA, and a voxel size of 25 μm for each sample to characterise the as-manufactured samples. The wall thickness method, applied through the ORS-Dragonfly 2022.2 software, was used to compare the as-manufactured samples with the CADs. As demonstrated by CAD metrological characterization, different statistical analysis of the data was performed based on the resulting histograms.

### 2.3. Microstructural and Mechanical Characterizations

The microstructural characterization is conducted through a light optical microscope (LOM Zeiss AxioPhot, Zeiss, Jena, Germany) and scanning electron microscopy (SEM JEOL IT300, JEOL, Tokyo, Japan) after standard metallographic preparation of the cross-section. Kroll’s reagent (1 mL of HF, 30 mL of HNO3, 85 mL of distilled water), prepared according to ASTM E407-07 [65], is used to highlight the microstructure of the cross-section. Three quasi-static compression tests for each geometry are carried out by means of a servo-hydraulic Instron 8516 testing machine (Instron, Norwood, MA, USA) equipped with an LVDT transducer to account for machine compliance. The machine is operated under stroke control, with a crosshead speed adjusted to achieve a strain rate between 10^−3^ s^−1^ and 10^−2^ s^−1^ at room temperature (20 ± 3 °C) according to ISO 13314:2011 [62]. The yield stress at 0.2% deformation is calculated from the quasi-static compression curves and the stabilised Young’s modulus for each geometry is calculated by applying five loading–unloading compression ramps between 20 and 70% of the yield stress [66,67]. Two tests for each geometry are carried out by using the same servo-hydraulic machine used for the quasi-static compression tests.

Fatigue testing is performed using the StepLab UDO 20 electro-actuated machine (STEP Lab, Treviso, Italy), equipped with the capability to configure testing frequencies. The tests are systematically conducted across various stress amplitudes, providing a comprehensive exploration of fatigue lives ranging from 10^4^ to 10^7^ cycles. Specimens undergo compression–compression fatigue testing at 90 Hz, with a stress ratio equal to R = 0.1 and a load cell of ±10 kN. The runout threshold is fixed at 10^7^ cycles. A minimum of twelve specimens for each geometry undergo testing, with three specimens tested at each of the four load levels. This approach is employed to establish the experimental Wöhler curve, providing a comprehensive understanding of the fatigue behaviour across different stress levels. The obtained Wöhler curves are then subjected to interpolation using a power law equation enriched with a constant term to capture the asymptotic trend displayed by the experimental data:

(4) $$\sigma_{max}=k_{1}+\frac{k_{2}}{N_{f}^{k_{3}}}$$

where *N_f_* represents the number of cycles to failure. The inclusion of runout tests in the fitting process aimed to provide a more robust and conservative estimation of the S-N curve. The coefficients k_i_ (i = 1, 2, 3) are evaluated by fitting σ_max_ vs. N_f_ curve on a logarithmic (log10) scale. The dispersion of the fatigue data is evaluated by calculating the estimated regression variance, assumed to be uniform across the entire fatigue life range and expressed in Equation (5).

(5) $$S^{2}=\frac{\sum_{i=1}^{n}{\left(\sigma_{max,i}-{\hat{\sigma }}_{max,i}\right)}^{2}}{n-p}$$

where *σ_max,i_* represents the i-th fatigue maximum data point, ${\hat{\sigma }}_{max,i}$ is its estimator, *n* is the total number of data points, and *p* denotes the number of parameters in the regression (in this case, *p* = 3).

### 2.4. Biological Assessment

#### 2.4.1. Cytotoxicity Assessment

To investigate the potential cytotoxicity of the material, a Lactate Dehydrogenase (LDH) cytotoxicity assay is conducted on samples using human lung fibroblast cells (MRC-5__ATCC, Manassas, VA, USA), following the ISO 10993-5 standard [68]. After the AM process, specimens are separated from the printing platform using the Electrical Discharge Machining (EDM) technique. Then, four replicates of each sample per condition (timepoint) are subjected to a careful cleaning process, involving ultrasonication in acetone, 95% ethanol, and distilled water for three cycles, each lasting 15 min. Finally, the sterilization process is carried out using an autoclave at 121 °C for 30 min.

Initially, cells are expanded in Minimum Essential Medium without phenol red (MEM__Gibco, Fisher Scientific Italy, Rodano, Italy) containing 10% Fetal Bovine Serum (FBS__Euroclone, Euroclone S.P.A., Pero, Italy), plus 1% L-glutamine (Euroclone, Euroclone S.P.A., Pero, Italy), 1% sodium pyruvate (Gibco, Fisher Scientific Italy, Rodano, Italy), 1% non-essential amino acids (Sigma Aldrich, Merck Italy, Milan, Italy), and 1% antibiotic/antimycotic (Euroclone, Euroclone S.P.A., Pero, Italy) in a humidified atmosphere at 37 °C with 5% CO_2_. After reaching the confluency, cells are seeded in a 96-well plate with a density of 5000 cells/well.

An indirect cytotoxicity assay under ISO 10993-5 is performed, using the above-mentioned culture medium with serum as the extraction vehicle. The samples are immersed in the extraction medium for 72 h at 37 °C in a volume calculated according to the ISO10993-12 for larger moulded items with thickness greater than 1 mm [69]. Subsequently, 100 µL of the medium is extracted and introduced to cells cultured in the 96-well plate at 70% of confluence. The positive control (CTRL+) involves fully lysed cells using a lysis buffer, which induces maximum LDH release. The negative control (CTRL−) is considered to be 100 µL of normal medium in contact with the cells. Wells containing culture medium without cells are considered blank samples. Cytotoxic effects are evaluated after 24 h and 48 h. At each time point, the medium from each well is collected and an LDH assay (CyQUANT™ LDH Cytotoxicity Assay kit, ThermoFisher Scientific, Fisher Scientific Italy, Rodano, Italy) is performed following the manufacturer’s instruction. Briefly, 50 µL of the medium of each sample is transferred into a transparent 96-well plate and mixed with 50 µL of the reaction mix. The plate is later incubated for 30 min in a dark environment. After this, the reaction 50 µL of Stop solution is added into each well to end the reaction. LDH release is quantified by measuring wavelength absorbance at 490 nm with a microplate reader (Tecan Infinite M200, Tecan Group Ltd., Männedorf, Switzerland).

#### 2.4.2. Cell Viability and Morphology

Human osteosarcoma cells (MG-63__ATCC, ATCC, Manassas, VA, USA) are used as a cell line for this experiment to investigate cells metabolic activity and adhesion over time. Samples are subjected to cleaning and sterilization before the experiment following the same procedure described for the cytotoxicity assessment. Cells are cultured in expansion medium composed of MEM (Gibco) with 10% FBS (Euroclone), as well as 1% L-glutamine (Euroclone), 1% sodium pyruvate (Gibco), 1% non-essential amino acids (Sigma Aldrich), and 1% antibiotic/antimycotic (Euroclone). After they reach the 70% of confluence, cells are detached and seed on each sample at a density of 4 × 10^4^ cells per sample in a seeding volume of 50 µL. The time points for cell culture experiment are 1, 3, and 7 days after seeding.

For metabolic activity, Alamar Blue assay is performed on 4 replicates per condition containing cells, plus 2 replicates as sample blanks without cells. Two-dimensional controls are represented by cells seeded on tissue culture plastic (TCP), and wells containing medium without cells serve as blank samples. In detail, at each time point, the samples are incubated in medium containing 10% resazurin stock solution (Resazurin Sodium Salt__Chemodex Ltd., Worksop, UK) for 3 h in a dark environment. Subsequently, 100 µL of supernatants of each well is added to a 96-well plate, and the absorbance of fluorescence intensity is read at 535 nm using 590 nm as a reference.

In the AlamarBlue assay, the ability of living cells to naturally reduce resazurin (Blue colour) to its fluorescent form, resorufin (Pink colour), is quantified by measuring the absorbance at a wavelength of 535 nm with the microplate reader. The objective of this assessment in our study is to compare the cell metabolic activity of various samples listed in Figure 2. A higher amount of cell activity corresponds to a higher absorbance.

Cell adhesion and morphology are investigated by imaging via confocal microscopy. Specifically, at each time point, samples are fixed using 4% paraformaldehyde (PFA__Sigma Aldrich, Merck Italy, Milan, Italy), washed twice in PBS, and the cell membranes permeabilised with a Triton X-100 (Sigma Aldrich) 0.2% (*v*/*v*) solution. After two additional washes in PBS, staining with 4′,6-diamidino-2-phenylindole (DAPI__Sigma Aldrich, Merck Italy, Milan, Italy) and OregonGreen (ThermoFisher Scientific, Fisher Scientific Italy, Rodano, Italy) is performed to visualize the cell nuclei and cytoskeleton, respectively. The morphology and adhesion behaviour of MG-63 cells on the samples in Figure 2 are examined using a laser scanning confocal microscope (LSCM, Nikon A1, Nikon, Tokyo, Japan) at day 1, 3, and 7 after seeding.

#### 2.4.3. Statistical Analysis

Repeated measurement analysis is used to validate the outcome of each assessment. Data are presented as the mean ± standard deviation and are analysed using a two-way ANOVA to calculate the difference significance. The difference is considered to be statistically significant if *p* < 0.05 (*p* value: * *p* < 0.05, ** *p* < 0.01, *** *p* < 0.001, and # represent **** *p* < 0.0001).

## 3. Results

### 3.1. 3D Metrological Characteristics

**Figure 5:**
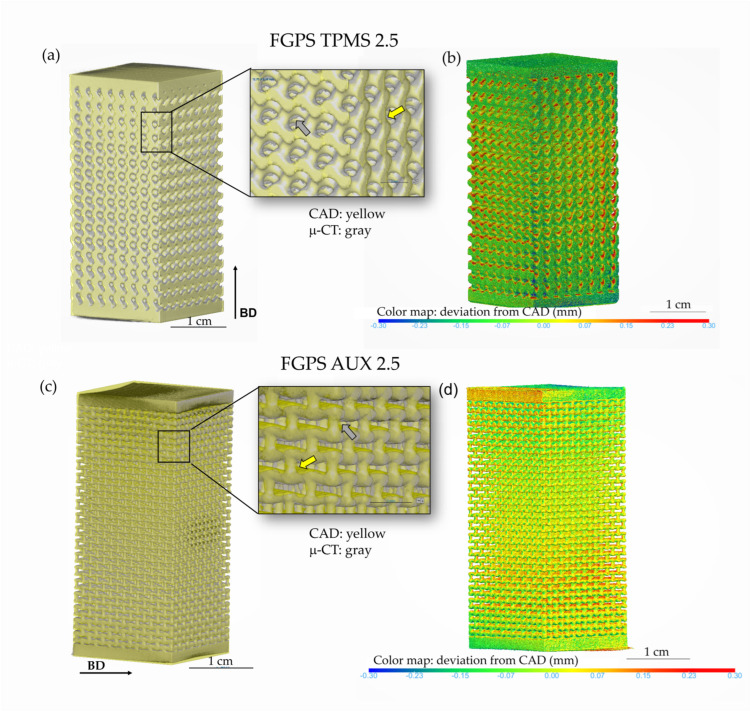
Visual comparison between μ-CT and CAD by means of Dragonfly overlapping function. (**a**) Overlap of μ-CT to CAD and detail to highlight the printability and (**b**) deviation color map of the FGPS-TPMS 2.5. (**c**) Overlap of μ-CT to CAD and detail to highlight the printability and (**d**) deviation color map of the FGPS-AUX 2.5.

μ-CT 3D images are used to evaluate the quality of the printed process in terms of dimensional deviation. The overlap of CAD (yellow) on the μ-CT image (grey) and the relative colour map of the deviation in case of TPMS-FGPS 2.5 and FGPS-AUX 2.5 are shown in Figure 5, respectively.

In the overlap details shown in Figure 5a (TPMS 2.5) and 5c (AUX 2.5), the excess of material, mainly located in the upper part of the pores in the as-manufactured sample, is indicated with a grey arrow, and the reduction in the strut and ligament thickness due to printed process is underlined by a yellow arrow in both FGPSs.

**Figure 6:**
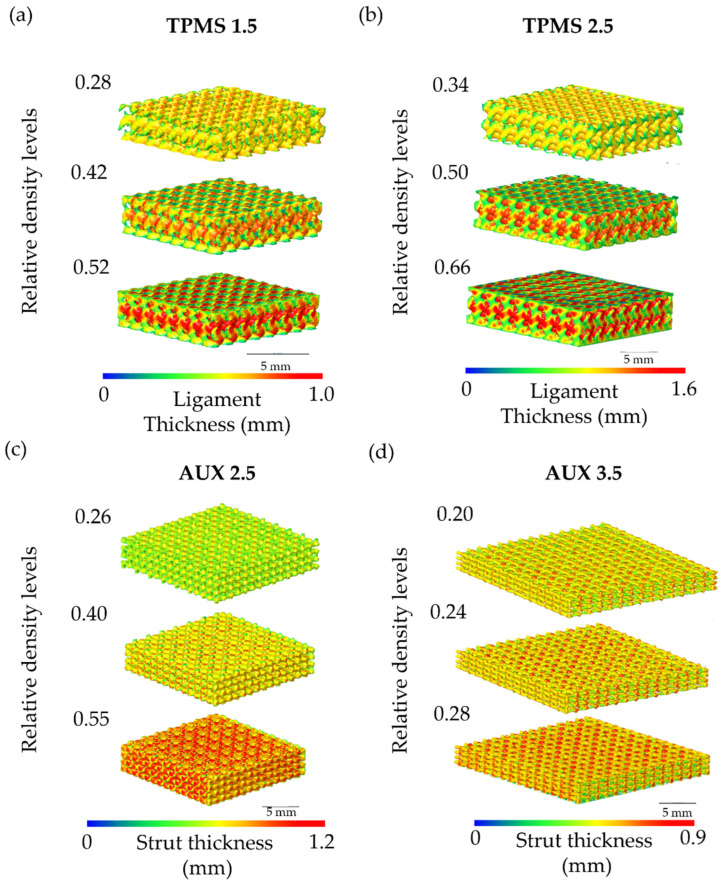
Color maps of the ligament thickness of the three relative density levels of (**a**) TPMS 1.5 and (**b**) TPMS 2.5. Strut thickness color maps of the three relative density levels of (**c**) AUX 2.5 and (**d**) AUX 3.5 obtained with Dragonfly software.

The alignment of the μ-CT image with the CAD reveals a strong correlation in strut or ligament inclination between the as-designed and as-manufactured samples, affirming the lack of geometric distortions caused by residual stresses. The as-manufactured pore size and strut or ligament thickness are measured using the wall thickness method for all the different geometries. Examples of the measurement in terms of strut for the auxetic re-entrant bow-tie structures and in terms of ligament thickness for the TPMS are shown in Figure 6.

**Figure 7:**
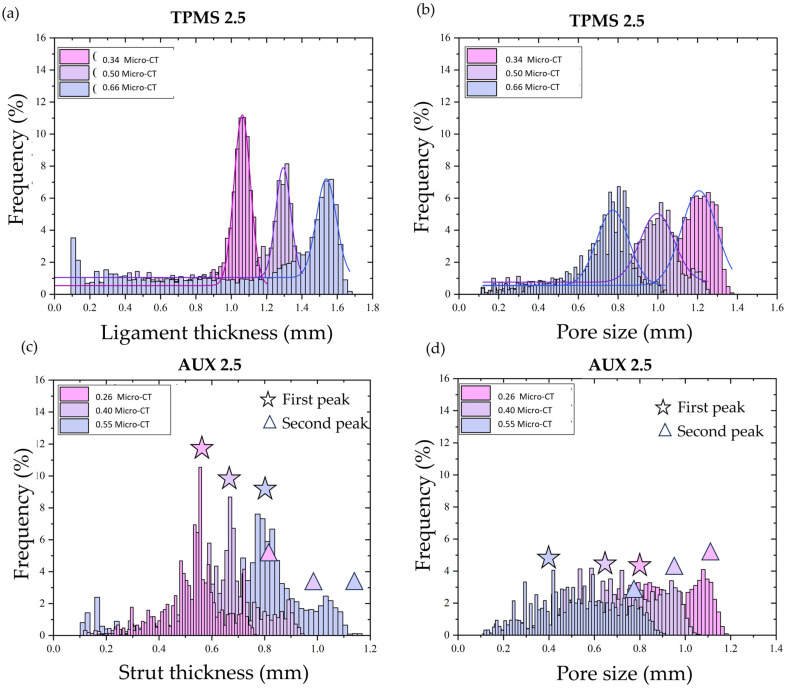
Data distributions of TPMS 2.5 in case of (**a**) ligament thickness and (**b**) pore size. Data distribution of AUX 2.5 in case of (**c**) strut thickness and (**d**) pore size.

The obtained data distribution of the ligament thickness and of the pore size for the TPMS structures could be well interpolated by means of Gaussian distribution curve as shown respectively in Figure 7a,b. However, in case of CAD, the data distributions of pore size and strut thickness of auxetic re-entrant bow-tie structures show two peaks as illustrated, respectively, in Figure 7c,d.

The peaks in the pore distribution are ascribed to the small pores located at the corners and to the larger pores in the middle of the auxetic re-entrant bow-tie structures. Conversely, the first peak in the strut thickness is associated to the strut size and the second peak to the nodes.

Summary of the data acquired for TPMS 1.5, TPMS 2.5, AUX 2.5, and AUX 3.5 are reported in Table 4.

**Figure 8:**
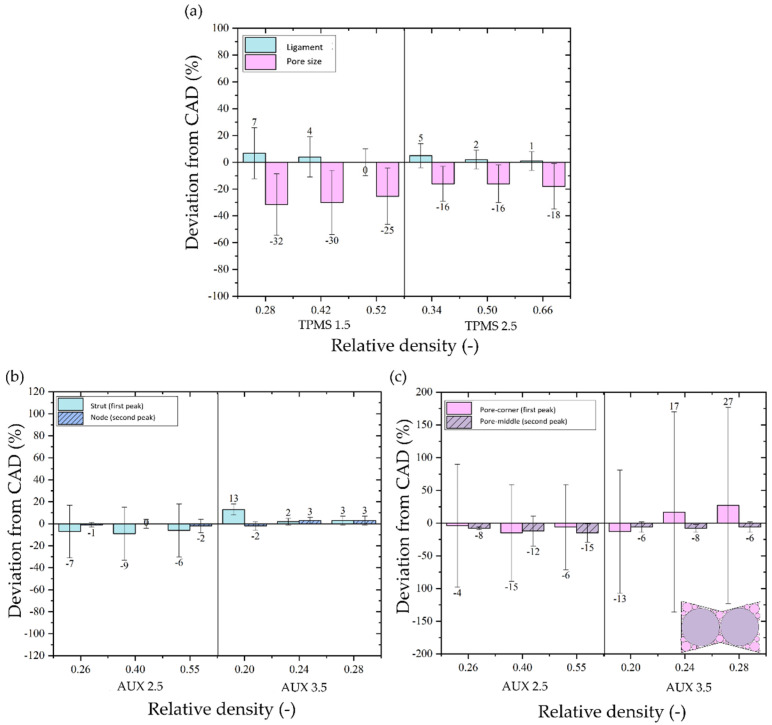
Deviation from CAD of the as-manufactured samples measured by means of the wall thickness method for the (**a**) TPMS 1.5 and 2.5 for all three relative density levels in terms of ligament and pore size. Deviation from CAD of the AUX 2.5 and AUX 3.5 considering the (**b**) strut and node thickness and (**c**) the pores at the corner and in the middle of the auxetic re-entrant bow-tie structures for all the relative density levels.

Comparing the CAD (Table 1) and μ-CT values (Table 4), it is possible to quantify the deviation of the as-manufactured samples from the design. Figure 8a shows the deviation from CAD considering the ligament thickness and the pore size of the two TPMS structures for all three relative density levels. Differently, the deviation from CAD in terms of the struts and nodes and the pores at the corner and middle of the auxetic re-entrant bow-tie structures for the three relative density levels are, respectively, illustrated in Figure 8b,c.

In all structures and for all relative density levels, the deviation from CAD is higher when considering the pore size rather than the ligament or strut thickness. Comparing the two TPMS structures, similar deviation from CAD is observed in the case of ligament thickness for all relative density levels, differently from pore size, where a higher deviation is observed in TPMS 1.5.

For the auxetic re-entrant bow-tie structures, an under-sizing of the strut and node is observed in AUX 2.5 with a maximum deviation of 10%. By contrast, a mix of under and over-sizing is observed in AUX 3.5. Regarding the pore size, the first peak refers to the small spheres (pink spheres in Figure 8c) that can be inscribed inside the corner of the auxetic re-entrant bow-tie structure and the second peak corresponds to the largest sphere (violet spheres) that can be inscribed inside the auxetic re-entrant bow-tie structures. In the case of small pores, the high standard deviation is ascribed to the high number of different sizes of spheres that can fill the corner as evident by looking at the data distributions in Figure 7c.

**Figure 9:**
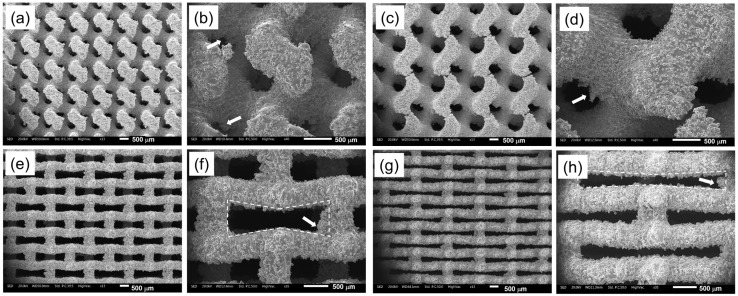
SEM micrograph of the overview and detail of the lattice structure of the lowest relative density level ρ_r1_ of (**a**,**b**) TPMS 1.5, (**c**,**d**) TPMS 2.5, (**e**,**f**) AUX 2.5 and (**g**,**h**) AUX 3.5.

To highlight the surface irregularity and the manufacturing accuracy, the SEM micrographs of the four lattice structures considering the lowest relative density level are reported in Figure 9.

Excess material on the upper part of the pore in the TPMS structures (Figure 9b,d) and at the corner of the auxetic re-entrant bow-tie structures (Figure 9f,h) are highlighted by white arrows inside the micrographs.

### 3.2. Microstructural and Mechanical Characterizations

**Figure 10:**
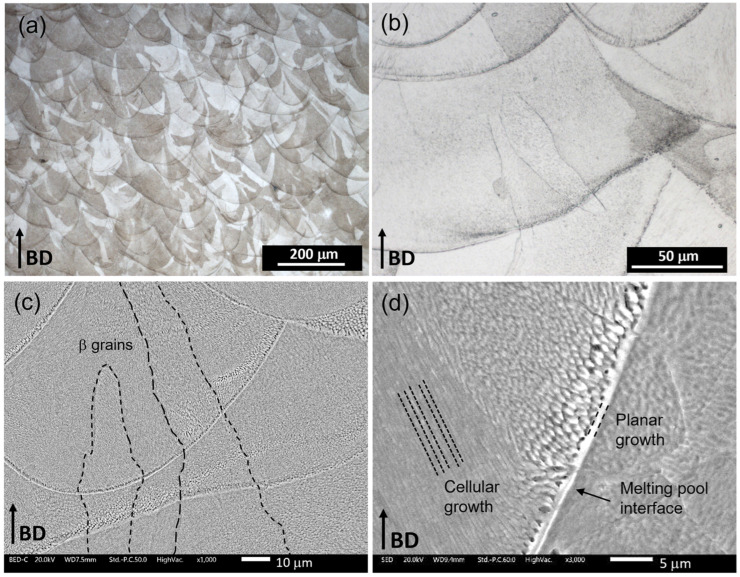
Details of the microstructure by means of (**a**,**b**) LOM analysis at low and high magnification and SEM details to highlight (**c**) the b grains and (**d**) the solidification morphology present inside all the four different lattice structures.

Micrograph details of the lattice structures are reported in Figure 10.

All samples exhibit the same microstructure. Therefore, Figure 10, showing the solid part of the FGPS-TPMS 2.5, represents the entire experimental campaign.

**Figure 11:**
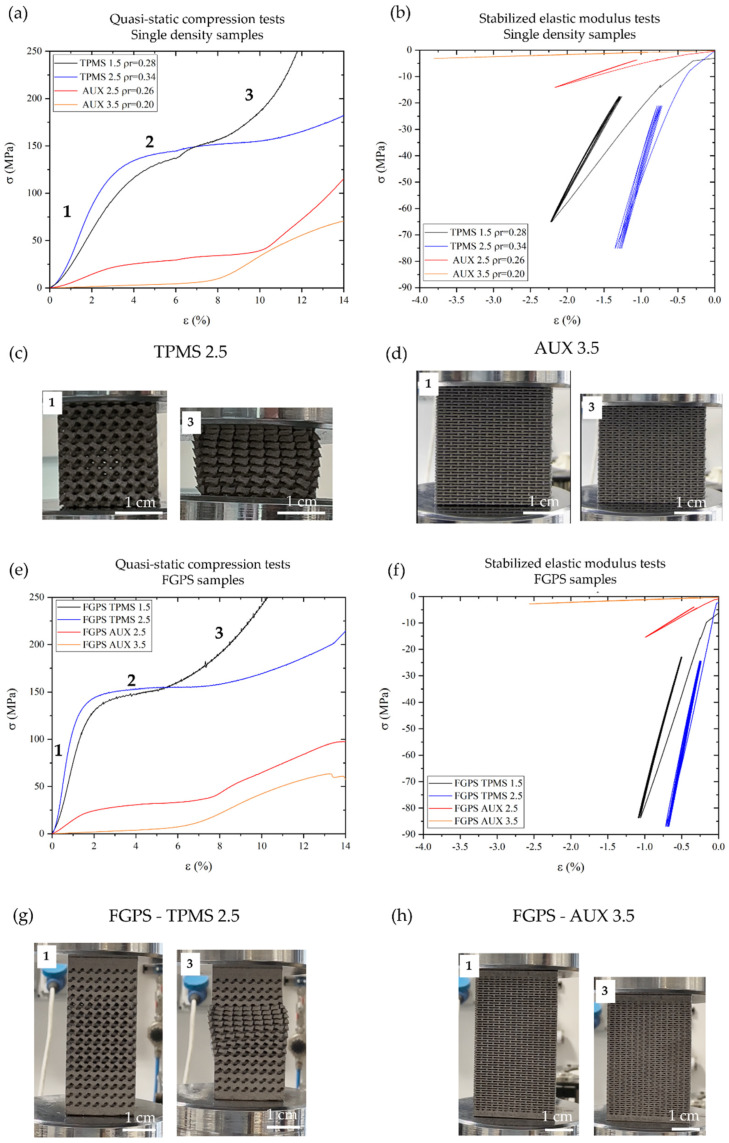
(**a**) Average quasi-static compression curves and (**b**) the average of five loading–unloading between 20% and 70% of the yield stress (σy) compression curves for each single density geometry. Details of the collapses observed at stages 1 and 3 of quasi-static compression curves of (**c**) TPMS 2.5 and of (**d**) AUX 3.5. (**e**) Average quasi-static compression curves and (**f**) average five loading–unloading between 20% and 70% σy compression curves for each FGPS geometry. Details of the collapses observed at stages 1 and 3 of quasi-static compression curves of (**g**) FGPS-TPMS 2.5 and of (**h**) FGPS-AUX 3.5.

The cross-section parallel to the building direction (BD), captured using LOM (Figure 10a), indicates a near-fully dense material and reveals the melt pool boundaries. At higher magnification (Figure 10b), the different solidification morphologies are visible due to the layer wise manufacturing process. The epitaxial growth of β grains, extending several millimetres in length, results from the partial remelting of previously consolidated layers, as demonstrated in earlier works. SEM micrographs at higher magnification emphasize the β grain boundaries (Figure 10c) and the solidification morphology aligned along the heat flow (Figure 10d). The average quasi-static and the average of five loading–unloading compression curves for single density and FGPS samples structures are summarised in Figure 11.

Figure 11a,b depicts average quasi-static compression curves and the average of five loading–unloading tests conducted at stress levels ranging from 20% to 70% of the yield stress for the constant density samples (the lowest relative density level). The quasi-static compression curves reveal three distinct regions: firstly, a linear elastic regime (1) extending up to the yielding point; secondly, a plateau regime (2) indicating structural collapse; and finally, the last stage (3) marked by densification, distinguished by a positive slope in the curve. Notably, AUX 3.5 shows an absence of a plateau regime, undergoing early densification compared to structural collapse.

Details of the deformation during stage 1 (linear elastic region) and stage 3 (densification regimes) are illustrated in Figure 11c,d for TPMS 2.5 and AUX 3.5, respectively.

To stabilise the mechanical behaviour, five loading–unloading cycles between 20% and 70% of the yield stress are conducted (Figure 11b). A change in slope is observed after the first loading–unloading cycle, which remains consistent throughout the subsequent four cycles. Higher slopes for the elastic modulus after achieving mechanical stabilisation following a preload are evident in all single density structures. Focusing on FGPS structures, designed to optimise the mechanical and biological response of the component, the effect of gradient porosity on the mechanical response is investigated through quasi-static compression tests and five loading–unloading tests at stress levels ranging from 20% to 70% of the yield stress, equal to those performed on single-density samples. The average curves of both quasi-static compression tests and stabilised elastic modulus tests on FGPS are reported in Figure 11e,f, respectively. As in the case of the single-density samples, a linear elastic regime (1), a plateau regime (2), and the final densification regime (3) are highlighted in the curves. The plateau regime is not observed in the FGPS AUX 3.5 since the densification of the auxetic re-entrant bow-tie structure occurs before the collapse as shown in the single-density sample. Details of the deformation occurring in the FGPS structures during stage 1 (the linear elastic region) and stage 3 (densification regimes) are illustrated in Figure 11g,h for FGPS-TPMS 2.5 and FGPS-AUX 3.5, respectively.

The average yielding stress ($\sigma_{y,0.2}$) and the average stabilised elastic modulus (E) for all single-density and FGPS geometries are summarised in Table 5. In addition, human bone values in terms of elastic modulus and yielding stress for cortical and cancellous parts are reported as benchmarks [70].

Comparing the elastic modulus of lattice structures with that of human bone, it is evident that both single density and FGPS auxetic re-entrant bow-tie and TPMS structures achieve elastic moduli close to those of human bone. Specifically, single and FGPS TPMS structures exhibit stiffness levels comparable to cortical bone. In contrast, single-density and FGPS auxetic re-entrant bow-tie structures demonstrate values similar to cancellous bone.

The AUX 3.5 exhibits the lowest elastic modulus, which is also accompanied by a yielding stress considered too low when compared to the less robust cancellous bone.

Comparing the single density with the FGPS mechanical responses, it is evident an increased yielding stress and elastic modulus by inserting a longitudinal porosity gradient in the TPMS structures. This does not occur in the auxetic re-entrant bow-tie structures, where the same low-yielding stress is obtained with and without the porosity gradient and a slight increase in the elastic modulus is achieved.

**Figure 12:**
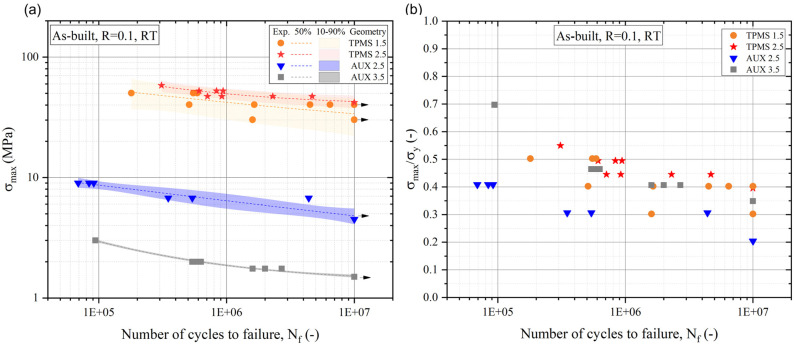
(**a**) Compression–compression fatigue Wöhler curves with both axes in log_10_ scale for all single-density geometries. Dotted lines represent 50% failure probability and solid lines refer to 10% and 90% failure probability. Arrows refer to runout tests. (**b**) Normalised compression–compression maximum fatigue stress for all single-density geometries.

The fatigue response of all the structures is evaluated in a compression–compression fatigue test on the more critical relative density level, namely, the lowest one. Results are depicted in Figure 12.

Fitting curves corresponding to 50% (dotted lines), 10%, and 90% (solid lines inside a coloured scatter band) failure probability evaluated by means of Equation (4) are plotted in Figure 12a.

To remove the influence of porosity, and to compare the fatigue response by only considering the cell type and dimension, normalization of the maximum fatigue strength with respect to the yield strength, which is also influenced by the porosity, was carried out as shown in Figure 12b.

The maximum fatigue strength at 10^7^ cycles, the standard deviation (S), and the normalised maximum fatigue strength at 10^7^ cycles and at 10^6^ cycles for comparison with the literature are summarised in Table 6.

A significantly high standard deviation is observed in TPMS 1.5 compared to TPMS 2.5.

From normalised data, better fatigue performance of the TPMS structures compared to the auxetic re-entrant bow-tie structure is identified. Considering the effect of unit cell size on TPMS, TPMS 1.5 exhibits lower fatigue performance compared to TPMS 2.5. In contrast, within different geometries in the auxetic structure, AUX 3.5 with a lower θ and a/b ratio shows higher normalised fatigue resistance compared to AUX 2.5.

**Figure 13:**
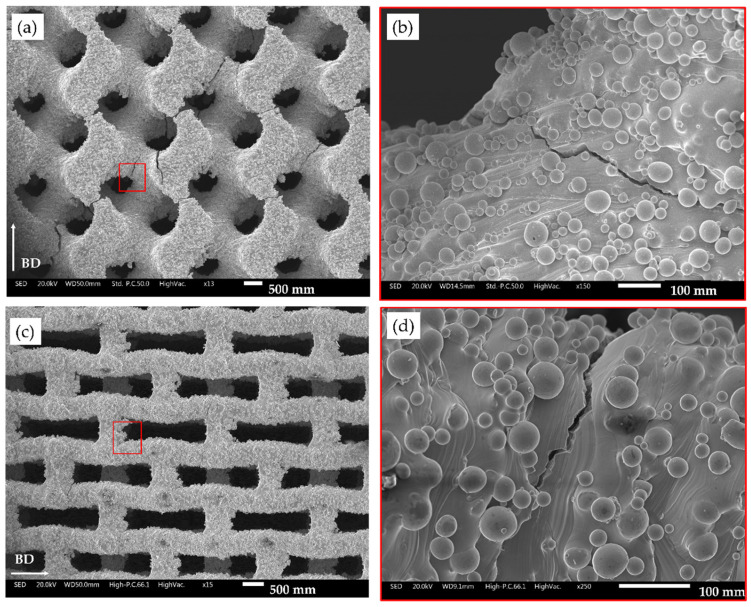
SEM micrographs of tested fatigue samples in case of (**a**) TPMS 2.5, (**b**) its detail of a nucleation fatigue crack site, (**c**) AUX 2.5, (**d**) its detail of a nucleation fatigue crack site.

Localization of the fatigue cracks on both auxetic re-entrant bow-tie and TPMS structures is reported in Figure 13.

It appears clear, observing the tested specimens that manufacturing-induced surface defects, such as partially melted particles and surface roughness, act as notches and promote crack nucleation. Fatigue cracks are identified in the presence of pre-existent manufacturing defects (Figure 13a,b), and, in auxetic re-entrant bow-tie cells, in defects close to the nodal junctions (Figure 13c,d).

### 3.3. Biological Assessment

#### 3.3.1. Cytotoxicity

**Figure 14:**
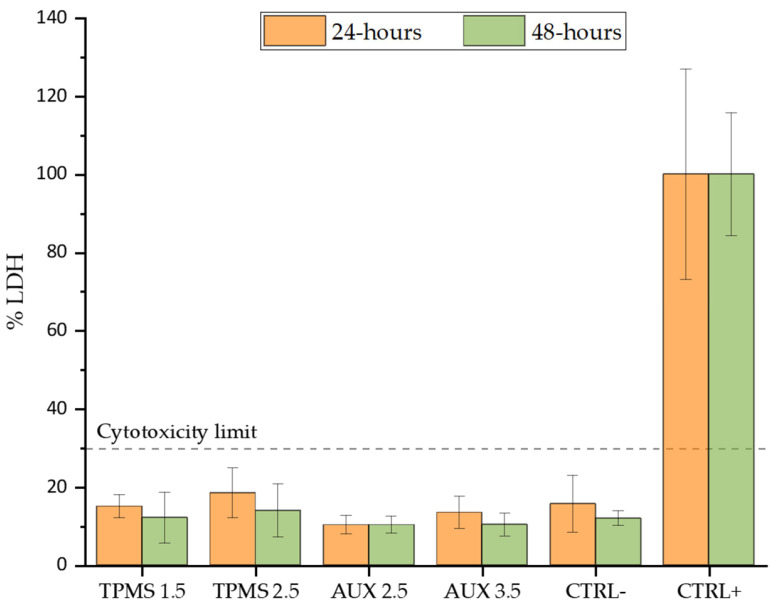
LDH release percentage of MRC-5 cells cultured on samples, after 24 and 48 h in a normal culture medium (abbreviations: CTRL−. tissue culture plastic, CTRL+. fully lysed cells).

In Figure 14, the outcomes of cytotoxicity assessment are displayed following 24 and 48 h of MRC5 cell culture using an indirect approach.

As already mentioned, the positive control represents 100% of LDH release. The results suggest that all tested conditions exhibit cell cytotoxicity below 20%, which is entirely in line with the LDH release observed in the negative control. In addition, no significant variation is detected comparing the different lattice structures.

#### 3.3.2. Cell Viability

**Figure 15:**
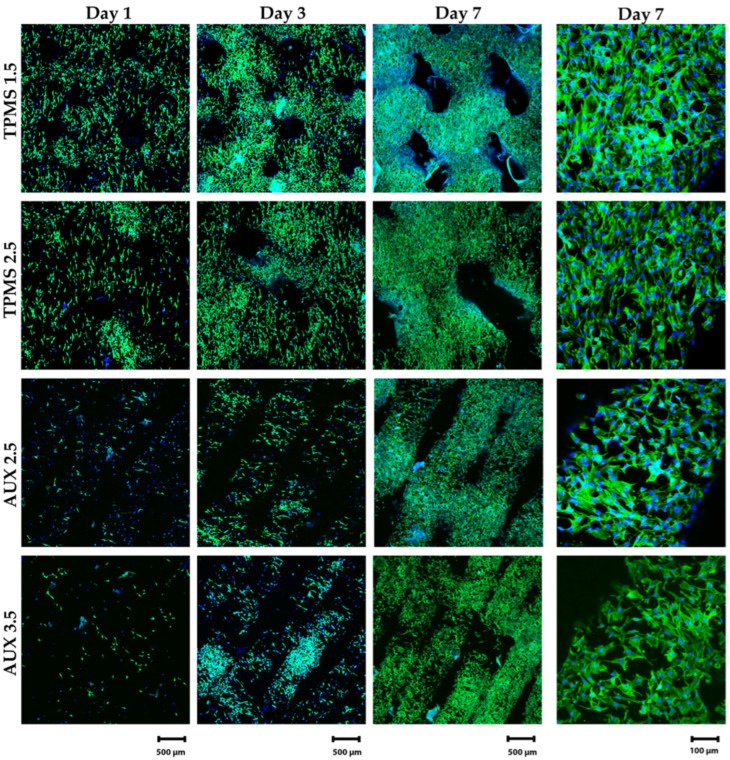
Confocal microscopy of MG-63 cell cultured on the samples (magnifications from left to right: 4×, 4×, 4×, 20×).

Figure 15 presents the confocal microscopy results of the MG-63 cell cultured on samples at two different magnifications.

On day 1, the AUX2.5 and AUX3.5 conditions exhibit a higher proportion of round-shaped cells compared to the TPMS1.5 and TPMS2.5 conditions, which display more elongated-shaped cells. Moreover, a lower cell density is observed on the surface of the AUX2.5 and AUX3.5 conditions, potentially due to the limited surface area on the microscopy depth range compared to the TPMS1.5 and TPMS2.5 samples.

By day 3, all samples demonstrate a notable improvement in cytoskeleton spreading patterns. As seen in Figure 15, on day 7, the MG-63 cells completely spread over the surface of all samples while proliferating on the designed geometries; however, the cell infiltration into the pores seems to be more pronounced in the TPMS1.5 and TPMS2.5 conditions. The presence of cell–cell interactions and the formation of cellular clusters are observed in all samples. Furthermore, as expected from the cytotoxicity results, all samples display satisfactory cytocompatibility, resulting in good cell adhesion and spreading over all the samples after 7 days.

**Figure 16:**
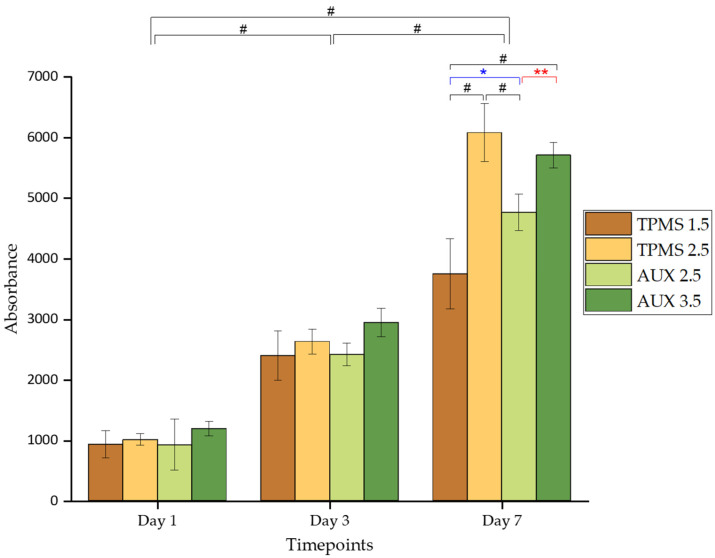
Metabolic activity of MG-63 cells cultured on samples at days 1, 3, and 7 after seeding (* *p* < 0.05, ** *p* < 0.01, ^#^ *p* < 0.0001).

Figure 16 presents the Alamar blue results for four conditions with a cell seeding density of 40 K.

The metabolic activity of cells cultured on various sample conditions was evaluated over a period of seven days. Initially, all groups exhibited comparable metabolic activity on the first day of culture with no significant differences observed. This similarity is likely due to consistent cell seeding efficiency across all samples, enabling meaningful comparisons at later time points.

By day 3, there was a consistent upward trend in absorbance across all groups, indicating an increase in cell activity over time. The substantial rise in absorbance from day 1 to day 7 suggests that both the material composition and geometric features of the samples supported cell viability throughout the experiment.

The consistent increase in metabolic activity observed from day 1 to day 7 across all samples in Figure 16 is in agreement with the confocal microscopy results shown in Figure 15. Over the course of the study, cells progressively grew and colonised the available surface area on each sample, thus resulting in an increase in metabolic activity.

Given the uniform cell seeding efficiency, the observed results can be attributed to the inherent cytocompatibility of the β-Ti21S material and the favourable geometric features of the samples. Despite variations in the cellular responses among the different structures, all samples demonstrated excellent biocompatibility, largely due to the superior properties of the β-Ti21S material, making it a reliable choice for biomedical applications.

Additionally, the porous nature of the samples provided a high surface area, creating an environment conducive to cell adhesion and proliferation. The surface topography provided by AM offered numerous anchoring points for cells to adhere and spread, and it plays a significant role in enhancing biological responses [71], as evidenced by the results of this study.

Notably, despite similar initial cell seeding densities, differences in metabolic activity among the samples may be attributed to variations in surface geometry and pore size. By day 7, AUX 2.5 and AUX 3.5 both exhibited more than five times higher metabolic activity compared to day 1. This substantial improvement can be attributed to the favourable curvature of the auxetic structure, which provides acute angle corners for enhanced cell attachment and growth [30,31,72,73]. Among the TPMS groups, TPMS 1.5 showed approximately 3.5 times increased metabolic activity by day 7, while TPMS 2.5 demonstrated a six-fold increase over the same period. The difference between TPMS samples may be attributed to the smaller pore size and reduced permeability of TPMS 1.5, as well as lower surface area for cellular activity. The increased surface area of TPMS 2.5 provides more extensive cell adhesion and colonization possibility and reduces the likelihood of cell confluency on the surface, thereby maintaining higher metabolic activity over time. In contrast, TPMS 1.5’s smaller pore size and lower permeability increase the risk of cellular overcrowding, potentially leading to reduced cell activity. Consequently, TPMS 1.5 exhibited the lowest metabolic activity on day 7, with a significant difference observed compared to the other three conditions. This reduced activity is believed to result from the smaller pore size of TPMS 1.5, which could limit permeability, likely hindering cell migration into the pores and limiting cellular colonization and metabolism at later time points [54].

Furthermore, TPMS 2.5 exhibited significantly higher metabolic activity than AUX 3.5, attributable to the unique geometry of the sheet-based TPMS structure. TPMS 2.5 offers a continuous, uninterrupted large surface area, and an interconnected pore structure that is conducive to cell adhesion. Moreover, the curvature profile of TPMS, which mimics trabecular bone, offers mechanical cues that create a biomimetic environment, promoting cytoskeletal expansion and cellular proliferation [74,75]. In contrast, auxetic structures, consisting of 2D layers with thin struts, sharp edges, and acute corners, stacked on top of one another, offering fewer connection points for cell migration, limiting the ability of cells to spread and crawl due to the lack of a continuous 3D surface [74,76]. This limitation likely accounts for the lower metabolic activity observed in AUX 2.5 compared to TPMS 2.5.

The author suggests that TPMS structures may be more suitable for promoting cell migration and proliferation due to their favourable geometric features. However, while TPMS demonstrates advantageous geometry compared to auxetic samples, differences in pore size significantly influenced cell viability. Specifically, TPMS 1.5, with its smaller pore size, was the least viable sample, whereas TPMS 2.5, with a more balanced pore size and geometry, exhibited one of the highest cell viability levels.

The statistical analysis using two-way ANOVA reveals a significant increase in metabolic activity across all groups from day 1 to day 7, supporting the confocal microscopy images analysis.

## 4. Discussion

### 4.1. Three-Dimensional Metrological Characteristics

The results from the μ-CT 3D images provide valuable insights into the printability and dimensional accuracy of the structures. The alignment of the μ-CT images with the CAD models shows that the printing process does not induce significant geometric distortions due to residual stresses, as evidenced by the strong correlation in strut and ligament inclination. The deviations observed in the pore size are higher than those in the ligament or strut thickness across all structures and relative density levels. This is justified considering the surface irregularity and the partially melted powder present on the surface of the strut. As demonstrated in previous works from the authors [18,59], these surface defects act drastically on the measurement of the pore size rather than on the ligament thickness using the wall thickness method. Based on this, to evaluate the printability of these lattice structures, we refer to the pore size deviation since it is more affected by the printed defects.

Comparing TPMS structures, TPMS 2.5 shows better printability than TPMS 1.5, with lower deviations in both ligament thickness and pore size. As demonstrated by Dallago et al. [19], an increased cell size from 1.5 mm to 3 mm resulted in an improved manufacturing accuracy of the lattice structure, confirming the best printability observed in TPMS 2.5 rather than TPMS 1.5.

In contrast, the auxetic re-entrant bow-tie structures exhibit better printability with under-sizing in AUX 2.5 and a mix of under and over-sizing in AUX 3.5, with deviations in strut and node thickness up to 10%.

The SEM micrographs confirm the presence of excess material, particularly in the upper parts of the pores for TPMS structures and at the corners of auxetic re-entrant bow-tie structures. These excesses contribute to the higher deviations observed, particularly in TPMS 1.5, where pore closure due to material excess significantly impacts dimensional accuracy.

In summary, while μ-CT and CAD comparisons indicate good overall printability, specific challenges such as surface irregularities and material excesses need to be addressed to enhance dimensional accuracy further. Future work could focus on optimising printing parameters and post-processing techniques to reduce these deviations and improve the fidelity of printed lattice structures to their CAD models.

### 4.2. Microstructural and Mechanical Characterisations

The as-printed microstructure exhibits epitaxial growth of β grains aligned along the heat flow direction. This phenomenon arises from the partial remelting of previously consolidated layers, effectively increasing β grain lengths to several millimetres, as documented in previous studies [9,77]. The orientation of the solidification structure, particularly the alignment of grains, is notably influenced by the local heat flow direction, which closely parallels the building direction, as illustrated in Figure 10c.

Further examination through SEM micrographs at higher magnification reveals a distinct evolution in the solidification structure. At the boundary of the melt pool, the structure appears planar, transitioning into a cellular pattern within the pool region, characterised by features approximately 0.5–1 μm in size. This transition occurs due to the destabilisation of the planar solidification front, primarily driven by constitutional undercooling. This phenomenon is attributed to a decreasing temperature gradient within the liquid ahead of the solid/liquid interface. When this gradient falls below a critical threshold, the solidification process shifts from planar to cellular [54].

The average quasi-static curves of both single density and FGPS structures illustrated in Figure 11a,c highlight the bending-dominated behaviour typical of all geometries, reflecting their respective cell topologies [22]. Specifically, the curves reveal early densification of the auxetic re-entrant bow-tie structure in the AUX 3.5 geometry, contrasting with its structural collapse and indicating the absence of a plateau regime.

To stabilise the mechanical behaviour, five loading–unloading cycles between 20% and 70% of the yield stress are performed on both single-density and FGPS structures, as depicted in Figure 11b,f. This procedure is particularly crucial due to the initial instability of lattice structures, especially at high porosity [78]. The instability manifests in the changing slope after the first loading–unloading cycle, which remains consistent in the subsequent four cycles. Higher slopes for the elastic modulus after achieving mechanical stabilisation following preload are observed in all single-density structures. During the initial loading cycle, a significant portion of the structure undergoes elastic loading; however, localised plastic deformation occurs at the ligament or strut junctions, leading to an uneven distribution of the strain field. Subsequent loading extends the elastic regime through strain hardening, resulting in increased stiffness [66].

The quasi-static compression tests on FGPS illustrate the impact of gradient porosity on the mechanical response of the component. The collapse progresses gradually, starting from less stiff (ρ_r1_) to stiffer levels (ρ_r3_), ultimately leading to the complete densification of the FGPS [22]. The five loading–unloading curves show an increased slope after the initial preload, confirming findings observed in single-density samples and underscoring the importance of stabilising the mechanical properties of lattice structures, even when gradient porosity is introduced.

**Figure 17:**
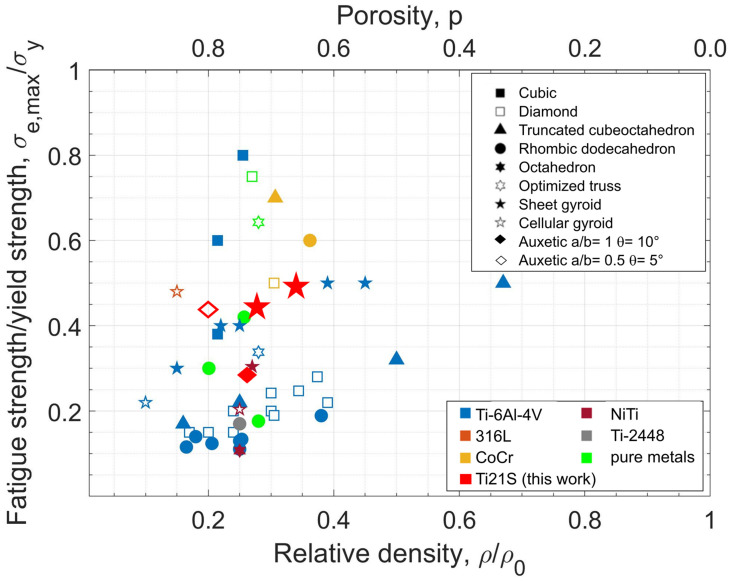
Comparison of normalised fatigue strength at 10^6^ cycles with literature data [22,27].

Considering the importance of structural stiffness to mitigate the stress shielding effect, the elastic moduli of lattice structures were evaluated in comparison to human bone. Both single-density and FGPS structures of auxetic re-entrant bow-tie and TPMS designs achieve elastic moduli that closely approximate those of human bone. Specifically, single and FGPS TPMS structures exhibit stiffness comparable to cortical bone, whereas single density and FGPS auxetic re-entrant bow-tie structures show values akin to cancellous bone. Notably, AUX 3.5 demonstrates the lowest elastic modulus, coupled with yielding stress deemed insufficient for withstanding biomechanical loads. Consequently, AUX 3.5 structures, in both single and FGPS configurations, are considered inadequate for mimicking human bone characteristics. Comparing the mechanical responses of single density and FGPS structures reveals that inserting a longitudinal porosity gradient in TPMS structures enhances both yielding stress and elastic modulus. This improvement indicates that the strength of TPMS structures increases with porosity gradient, although less stiff levels still dominate the overall mechanical response. In contrast, the insertion of a porosity gradient in auxetic re-entrant bow-tie structures does not result in a significant increase in yielding stress or elastic modulus. This outcome may be attributed to the inherent compliance of the selected auxetic re-entrant bow-tie structures [28]. This discussion underscores the critical role of structure design and porosity gradient in achieving desired mechanical properties comparable to human bone, emphasising the need for careful material selection and structural optimisation in biomedical applications. With the aim to replace and/or repair human bone, the fatigue response of the selected lattice structure is of primary importance due to the cyclic nature of the loads applied during the patient’s everyday life. As proven in [22], a decreased relative density and consequently increased porosity leads to a decreased fatigue resistance. The compression–compression fatigue tests highlight a very high standard deviation in TPMS 1.5 compared to TPMS 2.5. This evidence can be explained considering the worst manufacturability detected through the metrological investigation [19]: more excess of material and surface irregularity justify a higher scatter in the fatigue resistance since more variation in the surface defects that act as a nucleation point for the fatigue crack, as shown in Figure 13a,b. As demonstrated by Yavari et al. [79], the compression–compression fatigue strength of porous metal materials is principally affected by cell type and porosity. The normalised fatigue strength relative to the yield strength, essential for comparing different cell types while mitigating the influence of porosity, demonstrates superior fatigue performance of TPMS structures compared to auxetic re-entrant bow-tie structures. The strut-based topology of auxetic re-entrant bow-tie cell is indeed detrimental to fatigue life compared with TPMS since the nodal locations can act as stress raisers that promote fatigue crack nucleation as shown in Figure 13c,d. Nevertheless, AUX 3.5 with lower θ and a/b shows a high normalised fatigue resistance compared with AUX 2.5. On the other hand, TPMS structures are characterised by higher normalised strength thanks to the TPMS lattice topology that is intrinsically without junctions [32,37]. The lowest fatigue performance obtained in TPMS 1.5 compared with TPMS 2.5 is attributed to the printed defects that introduce intensification factors inside the geometries decreasing the fatigue life of the structures. The normalised fatigue resistance at 10^6^ cycles of this work can be added to the collected literature fatigue resistance data reported in the review of Benedetti et al. (Figure 17) [22].

**Figure 18:**
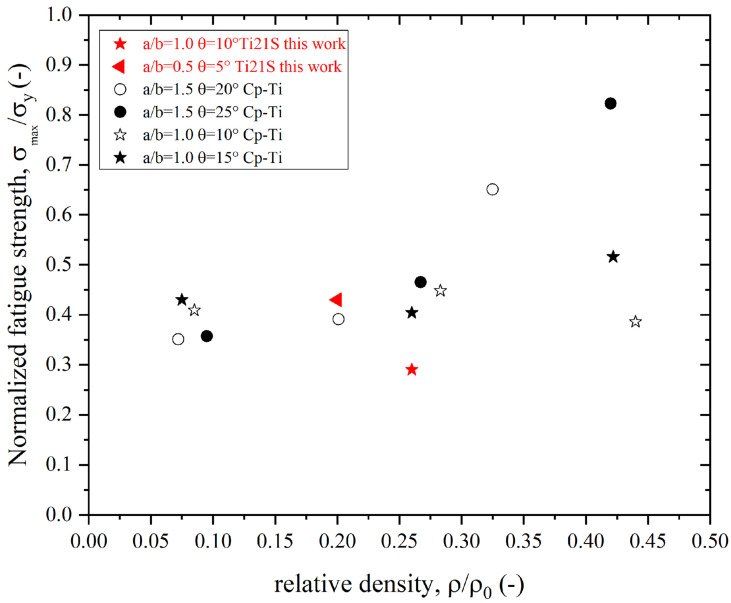
Data of normalised fatigue resistance of different auxetic re-entrant bow-tie structure with different a/b and θ from Kolken et al. [27] and from this work.

While the fatigue strength data are normalised with respect to the corresponding yield strength, a noticeable decrease in fatigue resistance is observed as porosity also increases in the analysed TPMS structures. This evidence suggests that the reduction in fatigue strength with increasing porosity is more prominent than the concurrent decrease in yield strength. Considering auxetic re-entrant bow-tie structures, the one with lower θ and a/b achieved higher fatigue resistance close to the sheet gyroid TPMS structure one. Very few authors have analysed the fatigue response of auxetic re-entrant bow-tie structures and no one considering the auxetic re-entrant bow-tie geometry with θ equal to 5° and a/b equal to 0.5 (AUX 3.5). Kolken et al. [27] evaluated the fatigue resistance of different auxetic re-entrant bow-tie geometries made from Cp-Ti with θ ranging from 10° to 25° and a/b from 1.0 to 1.5 with different porosity. Fixing the a/b ratio, they observed a decreased normalised fatigue strength at 106 cycles increasing the re-entrant angle θ from 10° to 15° (in case of a/b = 1.0) and from 20° to 25° (in case of a/b equal to 1.5) with similar relative density as shown in Figure 18.

Plotting the normalised fatigue data of this work regarding auxetic re-entrant bow-tie structures with the results obtained by Kolken et al. [27] it is possible to highlight the lower normalised fatigue resistance obtained in our work in the case of auxetic re-entrant bow-tie with a/b equal to 1 and θ equal to 10°. This could be justified considering the lower layer thickness of 30 μm used for their work rather than the 60 μm of this study used to increase productivity. It is well known that an increased powder layer thickness to increase productivity is usually associated with a decrease in accuracy and enhanced surface roughness that drastically affects the final mechanical performances [80,81,82]. In contrast with our result, Antonio Cutolo et al. [83] observed that even with a less accurate printability of the structure by increasing powder layer thickness from 30 to 60 μm no significant variation in fatigue resistance was observed. They specify that the increase in powder layer thickness must be associated with a proper optimisation of the other print parameters to not affect the mechanical behaviour.

### 4.3. Biological Assessment

Considering the cytotoxicity test, the observed cytotoxicity levels for all samples fall within non-toxic thresholds according to ISO 10993-5, being set at 30% [68].

These results confirm that Ti-21S is not cytotoxic, not only in bulk condition as demonstrated in a previous work [9], but also in lattice structures, where the surface area is larger compared to the bulk material, with no significant influence from the unit cell geometry and porosity.

Statistical analysis using two-way ANOVA confirmed a significant increase in metabolic activity across all groups from day 1 to day 7, supporting the qualitative analysis from confocal microscopy images. These results highlight the complex relationship between material geometry, pore size, and cellular response, crucial for the design and optimisation of biomaterials for tissue engineering applications.

The AM technique used in this study to prepare the samples significantly influenced the biological assessments. The samples were used in their as-built condition without undergoing any surface treatments. While this may pose challenges for the fatigue life of the implant, it can be advantageous for cellular attachment due to the increased surface roughness and the presence of partially melted powders on the surface, which provide additional anchoring points for cells [71]. This could be a reason why all samples exhibited significant cytocompatibility over the seven-day period.

However, additive manufacturing also introduces certain limitations to the structures. For example, defects such as incomplete fusion or pore occlusion can arise in samples with very small pore sizes or thin struts. This issue was observed in the TPMS 1.5 samples in this study, where poor printability led to clogged pores and reduced permeability, adversely affecting the biological assessments. The presence of such defects can completely or partially block pores, impeding bone ingrowth [71]. Therefore, it is crucial to consider these limitations and to conduct future in vivo studies to evaluate the real-world performance of these structures.

Another critical aspect of AM is the difference in surface roughness between upward-facing and downward-facing regions of the printed samples, which can influence cellular responses and growth efficiency. In this study, efforts were made to minimise this variability by using only the upward-facing sides of the samples. However, it is important to note that strut-based structures, such as the auxetic designs used in this study, are more prone to defects compared to TPMS structures, which tend to exhibit fewer printing inconsistencies [71]. These disparities make direct comparisons between the two geometries more challenging.

The observed differences in metabolic activity between samples with varying pore sizes align with previous findings by Ran et al. [45], who reported superior osteoblast cell viability in 3D-printed porous Ti6Al4V implants with larger pore sizes, despite better initial cell seeding efficiency in smaller pores. This underscores the critical roles of pore size, porosity, and permeability in influencing cell behavior and metabolic activity over extended periods. Permeability, in particular, plays a central role in cell proliferation, as greater permeability supports enhanced nutrient and waste transport. In this study, over time, samples with higher permeability demonstrated better cell survival, consistent with findings by Zhang et al. [54]

Additionally, the differences in cell behaviour observed on various geometries can be attributed to the distinct surface areas available for cell attachment and migration. Literature highlights that TPMS structures, with their large surface area and curvature profiles resembling trabecular bone, provide superior geometrical cues for cells to crawl and extend their cytoskeleton [75]. This phenomenon was also evident in the results of this study.

In contrast, auxetic structures predominantly offer thin struts and limited points of contact, which may restrict cell attachment and spreading. This structural limitation likely accounts for the overall reduced cell activity observed on auxetic samples compared to TPMS structures [75,76]. However, there is evidence supporting the effectiveness of auxetic geometries for cell adhesion, particularly due to the corners of negative curvature, which enhance cell attachment [30,31,72,73].

Overall, TPMS structures outperformed auxetic geometries, demonstrating superior support for cell proliferation and metabolic activity. In this study, the thin struts of the auxetic structures may have reduced the surface area available for cell growth. Adjusting the strut thickness could potentially improve cell activity and make results more comparable to TPMS samples.

## 5. Conclusions

This study investigated the manufacturability and quasi-static mechanical responses of two auxetic re-entrant bow-tie structures, namely, AUX 2.5 (with a unit cell size of 2.5 × 2.1 mm, a/b ratio of 1.0, θ equal to 10°) and AUX 3.5 (featuring a unit cell size of 3.5 × 1.45 mm, a/b ratio of 0.5, θ equal to 5°), alongside two TPMS structures—TMS 2.5 (unit cell size of 2.5 mm) and TPMS 1.5 (unit cell size of 1.5 mm)—all fabricated using laser powder bed fusion in β-Ti21S alloy. The structures exhibit a functionally graded porous design optimised for both mechanical and biological requirements. Furthermore, the study explored the quasi-static and fatigue mechanical responses of the structures at the lowest relative density level, aiming to further decrease stiffness. This contrasts with the focus on biological assessments at the highest relative density level, where the optimal pore size for bone attachment is characterised. In summary, the key findings of the research are as follows.

- Micro-CT scan analysis highlights the worst printability of TPMS 1.5, with an under sizing of the pore size around 30%, and a good correlation between CAD and as-manufactured samples in the case of TPMS 2.5, AUX 3.5, and AUX 2.5 with a maximum under-sizing of around 18%, considering all three relative density levels.
- The elastic modulus of all four lattice structures, under both single and functionally graded porous structures (FGPS) conditions, closely approximates that of human bone. Specifically, the single and FGPS TPMS structures exhibit stiffness within the range of cortical bone, while the single density and FGPS auxetic re-entrant bow-tie structures display values akin to cancellous bone. Notably, the AUX 3.5 stands out with the lowest elastic modulus, coupled with yield stress deemed insufficient for withstanding biomechanical loads. When comparing single density with FGPS mechanical responses, the dominance of less stiff levels in the structures’ mechanical behaviour is evident. An enhancement in strength, resulting in an increase in elastic modulus, is achieved by incorporating a porosity gradient within the TPMS geometries. However, this improvement is not observed in auxetic re-entrant bow-tie structures, where the same low-yielding stress is obtained with or without the porosity gradient. Additionally, a slight increase in the elastic modulus is noted, attributed to the excessively high compliance of the selected auxetic re-entrant bow-tie structures.
- The TPMS 1.5 exhibits the lowest maximum fatigue resistance, reaching 10^7^ cycles with a notable scatter deviation (33.9 ± 34.2 MPa), in contrast to the TPMS 2.5 (42.8 ± 6.1 MPa). This disparity is attributed to the TPMS 1.5’s challenging printability, leading to a higher incidence of defects within the structure. The pronounced difference is further highlighted when considering the normalised fatigue resistance, where the TPMS 1.5 registers a value of 0.3 at 10^7^ cycles compared to the TPMS 2.5, which is closer to 0.4. This emphasises the impact of printability issues on structural integrity and fatigue performance, particularly evident in the higher defect prevalence within the TPMS 1.5.
- Even with a high normalised fatigue resistance at 10^7^ cycles, the extremely low fatigue of 1.5 MPa renders the AUX 3.5 unsuitable for use in femoral implants, despite its low stiffness approaching that of cancellous bone (0.08 GPa). In contrast, the AUX 2.5 demonstrates a maximum compression fatigue resistance of approximately 5 MPa at 10^7^ cycles and an elastic modulus close to 1 GPa comparable with the mechanical response of the cancellous bone. This positions the AUX 2.5 as a favourable choice for reducing stiffness while maintaining strength levels aligned with those of human bone.
- The release of LDH from MRC-5 fibroblast cells, measured after 24 and 48 h of exposure to sample extractions, remained within the non-toxic range. This indicates that additively manufactured β-Ti21S samples featuring auxetic re-entrant bow-tie and TPMS lattice structures are non-toxic to human cells.
- Confocal microscopy analysis revealed robust cell adhesion and spreading across all samples. On the initial day, TPMS samples displayed a more elongated cytoskeleton compared to auxetic re-entrant bow-tie structures. By day 7, all samples exhibited complete coverage by cells, with well-spread cellular morphology indicating favourable cell–surface interaction and proliferation.
- Alamar Blue assay revealed a consistent upward trend in metabolic activity from Day 1 to Day 7, aligned with cell morphology findings. The majority of samples demonstrated an approximately five-fold increase in metabolic activity after 7 days, with the notable exception of TPMS 1.5, which exhibited a less than four-fold increase compared to the initial day. This difference can be attributed to TPMS 1.5’s smaller pore size range in comparison to the other samples, as well as a notable deviation between the real and designed pore size. This outcome is likely the result of pore size under-sizing and diminishing interconnectivity between pores in TPMS 1.5 due to the poor printability and less accurate manufacturing of specimens. Given the critical importance of efficient fluid permeability for cell viability, TPMS 1.5 did not demonstrate this factor as effectively as the other samples.
- Compared to the AUX samples, TPMS 2.5 exhibited higher metabolic activity in the AlamarBlue assay. This can be attributed to its continuous, large surface area, and favourable curvature that closely mimics trabecular bone. These features create an optimal environment for cells to adhere, grow, and migrate effectively.

## Figures and Tables

**Table 1:** CAD parameters evaluated by means of ORS-Dragonfly 2022.2 software.

	TPMS 1.5		TPMS 2.5		AUX 2.5		AUX 3.5	
ρ_r_	Ligament (mm)	Pore (mm)	Ligament (mm)	Pore (mm)	Strut Node (mm)	Pore_corner_ Pore_middle_ (mm)	Strut Node (mm)	Pore_corner_ Pore_middle_ (mm)
**1**	0.59	0.95	1.01	1.44	0.57 ± 0.03 0.73 ± 0.01	0.82 ± 0.46 1.18 ± 0.01	0.48 ± 0.01 0.62 ± 001	0.76 ± 0.43 1.48 ± 0.02
**2**	0.73	0.80	1.27	1.19	0.70 ± 0.01 0.89 ± 0.00	0.71 ± 0.29 1.05 ± 0.04	0.54 ± 0.01 0.66 ± 0.01	0.39 ± 0.24 1.45 ± 0.02
**3**	0.87	0.67	1.52	0.94	0.85 ± 0.10 1.05 ± 0.01	0.52 ± 0.20 0.92 ± 0.01	0.59 ± 0.01 0.72 ± 0.01	0.34 ± 0.18 1.39 ± 0.02

**Table 2:** CAD geometrical details of the single-density mechanical specimens with the lowest relative density level.

Structure	Dimension of Specimens			Relative Density	Ligament [TPMS] Strut/Node [AUX]	Pore [TPMS] Pore_corner_/Pore_middle_ [AUX]
	l (n Cell)	w (n Cell)	h (n Cell)	ρ_r_ (-)	(mm)	(mm)
**TPMS 1.5**	8	8	8	0.28	0.59	0.95
**TPMS 2.5**	8	8	8	0.34	1.01	1.44
**AUX 2.5**	8	8	10	0.26	0.57 ± 0.03 0.73 ± 0.01	0.82 ± 0.46 1.18 ± 0.01
**AUX 3.5**	8	8	20	0.20	0.48 ± 0.01 0.62 ± 0.01	0.76 ± 0.43 1.48 ± 0.02

**Table 3:** Ti-21S chemical composition and Mo equivalent.

Element	Ti	Mo	Al	Nb	Si	O	H	N	Fe	Ni	Other	MoE
wt.%	Bal.	14.9	3.24	2.96	0.22	0.11	ND	<0.010	0.048	-	<0.40	12.6

**Table 4:** Summary of the dimensions of the as-printed samples (based on μ-CT images), specifically pore size, ligament, and strut thickness, for TPMS 1.5, TPMS 2.5, AUX 2.5, and AUX 3.5 at the three corresponding relative density levels.

	TPMS 1.5		TPMS 2.5		AUX 2.5		AUX 3.5	
ρ_r_	Ligament (mm)	Pore (mm)	Ligament (mm)	Pore (mm)	Strut Node (mm)	Pore_corner_ Pore_middle_ (mm)	Strut Node (mm)	Pore_corner_ Pore_middle_ (mm)
**1**	0.63 ± 0.11	0.65 ± 0.22	1.06 ± 0.09	1.21 ± 0.18	0.53 ± 0.11 0.72 ± 0.01	0.79 ± 0.33 1.08 ± 0.01	0.54 ± 0.02 0.61 ± 002	0.66 ± 0.34 1.39 ± 0.06
**2**	0.75 ± 0.11	0.56 ± 0.19	1.29 ± 0.09	1.00 ± 0.17	0.64 ± 0.16 0.89 ± 0.04	0.60 ± 0.28 0.92 ± 0.21	0.55 ± 0.01 0.67 ± 0.02	0.46 ± 0.32 1.34 ± 0.06
**3**	0.87 ± 0.09	0.50 ± 0.14	1.54 ± 0.11	0.77 ± 0.16	0.80 ± 0.16 1.03 ± 0.10	0.49 ± 0.15 0.78 ± 0.12	0.61 ± 0.01 0.74 ± 0.03	0.43 ± 0.29 1.31 ± 0.08

**Table 5:** Summary of the quasi-static compression properties, namely, yielding stress and stabilised elastic modulus for single density and FGPS structures and compression properties along the longitudinal axis of the human bone, namely, the cortical and cancellous bones.

LATTICE STRUCTURE (Compression Properties)					
Structure Single Density	σ_y_ (MPa)	E (GPa)	Structure FGPS	σ_y_ (Mpa)	E (GPa)
**TPMS 1.5** **ρ** **_r1_** **= 0.28**	100.3 ± 3.1	5.69 ± 0.68	**TPMS 1.5**	123.0 ± 9.5	11.08 ± 0.67
**TPMS 2.5** **ρ** **_r1_** **= 0.34**	106 ± 3.6	5.83 ± 0.89	**TPMS 2.5**	125.3 ± 0.6	13.94 ± 0.64
**Aux 2.5** **ρ** **_r1_** **= 0.26**	22 ± 1	0.96 ± 0.02	**Aux 2.5**	23.0 ± 0.4	1.70 ± 0.05
**Aux 3.5** **ρ** **_r1_** **= 0.20**	4.3 ± 0.1	0.08 ± 0.01	**Aux 3.5**	3.8 ± 0.1	0.10 ± 0.00
**HUMAN BONE (Compression properties along the longitudinal axis)** [70]					
**Cortical**	131–224	6–20	**Cancellous**	5–10	0.08–4

**Table 6:** Principal results of the fatigue tests on all single-density geometries.

Geometries	σ_max_ (10^7^ Cycles) (MPa)	S (MPa)	σ_max_/σ_y_ (10^7^ Cycles) (-)	σ_max_/σ_y_ (10^6^ Cycles) (-)
**TPMS 1.5** **ρ** **_r1_** **= 0.28**	33.9	34.2	0.30	0.42
**TPMS 2.5** **ρ** **_r1_** **= 0.34**	42.8	6.1	0.40	0.47
**Aux 2.5** **ρ** **_r1_** **= 0.26**	4.8	0.5	0.20	0.29
**Aux 3.5** **ρ** **_r1_** **= 0.20**	1.5	0.0	0.35	0.43

## Data Availability

The original contributions presented in the study are included in the article, further inquiries can be directed to the corresponding author.
